# Experimental study of beam distortion due to fiducial markers during salvage HIFU in the prostate

**DOI:** 10.1186/s40349-018-0109-3

**Published:** 2018-03-22

**Authors:** Marina Bakaric, Eleanor Martin, Panayiotis S. Georgiou, Benjamin T. Cox, Heather Payne, Bradley E. Treeby

**Affiliations:** 1 Department of Medical Physics and Biomedical Engineering, University College London, Gower Street, London, WC1E 6BT UK; 2 Department of Oncology, University College London Hospitals, 235 Euston Road, London, NW1 2BU UK

**Keywords:** HIFU, Prostate cancer, Salvage treatment, EBRT, Fiducial marker, Feasibility, Phantom

## Abstract

**Background:**

Prostate cancer is frequently treated using external beam radiation therapy (EBRT). Prior to therapy, the prostate is commonly implanted with a small number of permanent fiducial markers used to monitor the position of the prostate during therapy. In the case of local cancer recurrence, high-intensity focused ultrasound (HIFU) provides a non-invasive salvage treatment option. However, the impact of the fiducial markers on HIFU treatment has not been thoroughly studied to date. The objective of this study was to experimentally investigate the effect of a single EBRT fiducial marker on the efficacy of HIFU treatment delivery using a tissue-mimicking material (TMM).

**Methods:**

A TMM with the acoustic properties of the prostate was developed based on a polyacrylamide hydrogel containing bovine serum albumin. Each phantom was implanted with a cylindrical fiducial marker and then sonicated using a 3.3 MHz focused bowl HIFU transducer. Two sets of experiments were performed. In the first, a single lesion was created at different positions along either the anteroposterior or left-right axes relative to the marker. In the second, a larger ablation volume was created by raster scanning. The size and position of the ablated volume were assessed using a millimetre grid overlaid on the phantom.

**Results:**

The impact of the marker on the position and size of the HIFU lesion was significant when the transducer focus was positioned within 7 mm anteriorly, 18 mm posteriorly or within 3 mm laterally of the marker. Beyond this, the generated lesion was not affected. When the focus was anterior to the marker, the lesion increased in size due to reflections. When the focus was posterior, the lesion decreased in size or was not present due to shadowing.

**Conclusions:**

The presence of an EBRT fiducial marker may result in an undertreated region beyond the marker due to reduced energy arriving at the focus, and an overtreated region in front of the marker due to reflections. Depending on the position of the targeted regions and the distribution of the markers, both effects may be undesirable and reduce treatment efficacy. Further work is necessary to investigate whether these results indicate the necessity to reconsider patient selection and treatment planning for prostate salvage HIFU after failed EBRT.

## Background

Prostate cancer is the most commonly occurring cancer in men [1]. External beam radiation therapy (EBRT) is frequently used as a definitive treatment for localised and locally advanced prostate cancer. Prior to commencing EBRT, permanent fiducial markers are commonly implanted into the prostate to increase the accuracy of treatment as part of image-guided radiotherapy (IGRT). These are used for localisation of the prostate, motion and deformation tracking, and as reference points for distance measurements [2, 3]. Despite the effectiveness of EBRT, cancer can reoccur locally in the prostate in up to 30% of patients [4]. In such cases, further local treatment using an alternative (salvage) therapy can be considered.

High-intensity focused ultrasound (HIFU) provides a non-invasive salvage treatment option for patients with recurrence after EBRT. During HIFU treatment, an ultrasound beam is generated with energy sufficient to induce tissue destruction within the focal volume by means of thermal, mechanical, and cavitation effects [5]. The ablated volume has a well-defined margin, which is one of the main advantages of HIFU therapy. HIFU treatment for prostate cancer can be delivered to the whole gland or focally to only the cancerous lesion within the prostate. The ablation is given in blocks in order to treat small volumes of the prostate sequentially, so as to limit damage to adjacent healthy tissue. Good local cancer control has been reported by several studies [4, 6–9].

However, an open question remains as to the impact of fiducial markers implanted during the EBRT procedure on the efficacy of post-EBRT salvage HIFU treatments. Typically, three gold fiducial markers in the shape of a cylinder (approximately 3 mm in length and 1 mm in diameter) are implanted in the prostate before the patient undergoes EBRT planning, and remain permanently in the prostate after the completion of the treatment [10]. As the characteristic acoustic impedance of the gold marker (*ρ*=19300 kg/m^3^, *c*=3240 m/s) is significantly higher than the impedance of the prostate tissue (*ρ*=1050 kg/m^3^, *c*=1578 m/s), this could potentially lead to distortion of the beam due to scattering or reflections when obstructed by the marker, compromising treatment efficacy.

Two previous studies have investigated the effect of low-dose brachytherapy seeds on HIFU treatment with mixed results [11, 12]. Brachytherapy seeds have a similar size and acoustic impedance to gold fiducial markers, thus the effect of the seeds on HIFU is likely to be similar. In 2003, a feasibility study for the treatment of prostate cancer using HIFU after brachytherapy failure was done by Seip et al. [11]. By performing in vivo canine experiments, they suggested that intraprostatic brachytherapy seeds have no impact on HIFU therapy. However, in 2007, Chapman et al. [12] performed lesioning experiments using *ex vivo* bovine liver and showed that seeds in the pre-focal region reduce HIFU lesion length and increase lesion variability, with the greatest effect when they are positioned close to the focus.

More recently, a series of simulations was used to investigate the impact of EBRT fiducial markers on HIFU treatment, using a numerical model of the prostate containing a single spherical or cylindrical fiducial marker [13]. The results indicated that placing a marker in the pre-focal region can induce a reduction in intensity of more than 30% of the homogeneous value, which will, in turn, cause a reduction in the volume of the ablated region. The simulations showed that the marker’s effect depends on its shape and distance from the focus of the transducer, with its impact significantly increasing when placed within 5 mm of the focus [13].

Most recently, a study was done investigating the effect of voxel masking in magnetic resonance-guided HIFU due to the presence of biopsy markers [14]. The study suggested that the markers had a minimal impact on the size of the target volume but that a smaller sonication on a marker might be more problematic.

The aim of the current study was to experimentally investigate the numerical results presented in [13]. Specifically, to produce thermal lesions in a tissue-mimicking material with and without a single EBRT fiducial marker obstructing the HIFU beam, and to investigate any changes in the size and position of the lesion due to the presence of the marker.

## Methods

### Phantom fabrication

A tissue-mimicking material (TMM) with the properties of the prostate was manufactured based on a polyacrylamide hydrogel (PAG) embedded with bovine serum albumin (BSA), as proposed by Lafon et al. [15]. BSA is a protein used as a temperature-sensitive indicator and thus shows a localised response to HIFU treatment in the form of an opaque lesion with a well-defined margin [16]. This TMM was chosen because its acoustic and thermal properties are similar to that of human soft tissue and can be tuned to match the desired properties [17–19]. The material is stable at high temperatures and pressures, and provides visible feedback of the lesion created with HIFU.

Previously, both the acoustic and thermal properties of BSA PAG have been characterised [15, 20]. Selected parameters are presented in Table 1, with the properties of prostate tissue shown for reference. While most of the TMM properties are close to those in prostate tissue, the measured attenuation coefficient of the TMM with either 9% BSA concentration [15] or 7% BSA concentration with increased acrylamide concentration and added glass beads [20] are below or well above the median value reported for the prostate. In order to suit the requirements for a prostate phantom, the recipe reported by Khokhlova et al. [21], who reported attenuation coefficient values in gel with 7% BSA of about one-third of the value in tissue, was modified as follows. First, the concentration of BSA was increased in order to achieve higher attenuation (it has previously been reported that the attenuation increases linearly with the concentration of BSA [15]). Second, the initial concentration of a 40% solution of acrylamide was increased to produce more stable phantoms, as this is what determines the hardness and crosslinking of the gel [22].

**Table 1:** Sound speed *c*, density *ρ*, impedance *z*, and acoustic attenuation coefficient *α* for standard BSA PAG ^*a*^, TM BSA PAG ^*b*^ with increased acrylamide concentration and added glass beads, and the newly developed BSA PAG in comparison to the values for the human prostate ^*c*^

Material	c (10^3^ m/s)	*ρ* (10^3^ kg/m^3^)	z (10^6^ kg/m ^2^/s)	*α* (dB/cm)	f (MHz)
BSA PAG	1.54 ± 0.01	1.04 ± 0.02	1.60 ± 0.01	0.58	3.5
TM BSA PAG	1.58 ± 0.01	1.06 ± 0.01	1.67 ± 0.01	1.91	3.5
New BSA PAG	1.55 ± 0.02	1.09 ± 0.02	1.70 ± 0.02	1.10	3.5
Prostate	1.58	1.05	1.66	0.5-1.5	3.5

*TM* Tissue-mimicking, *BSA* Bovine Serum Albumin, *PAG* Polyacrylamide Hydrogel

^a^Lafon et al. [15]

^b^Choi et al. [20]

^c^Duck [24]; Worthington et al. [30]

The final recipe for a 200 ml phantom with 14% BSA concentration was as follows:

1. 14.000 g of BSA was dissolved in 143.22 ml of degassed distilled water (71.61%) and then placed in a vacuum chamber for 1 hour.
2. 40.00 ml of a 40% (w/v) solution of acrylamide/bis 19:1 (20%) was then added to the mixture, followed by 20.00 ml of 1M TRIS buffer pH 8 (10%), and 1.68 ml of APS (0.84%).
3. The entire solution was placed in a vacuum chamber for 1 hour for additional degassing.
4. Polymerization was finalised by adding 0.10 ml of degassed TEMED (0.05%).
5. The solution was then transferred into 5 ml Leur Slip Terumo syringes (used as moulds), and allowed to polymerise under airtight conditions at a temperature of 2°C overnight (the polymerization is highly exothermic and can reach temperatures up to 80°C, thus refrigeration is necessary)
6. After fabrication, the phantoms were stored in a vacuum-sealed bag and refrigerated at 2°C to prevent microbial invasion.

### Phantom characterisation

**Fig. 1:**
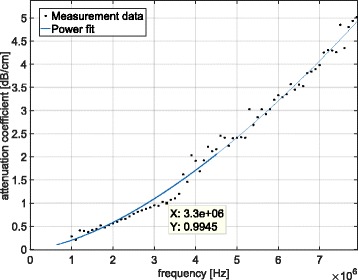
Measured acoustic attenuation coefficient in the tissue-mimicking phantom made from a polyacrylamide hydrogel containing bovine serum albumin. The data points represent the experimental data, and the solid line is a power law fit with parameters 0.156*f*^1.55^

In order to characterise the acoustic properties of the BSA PAG phantoms, two slabs with dimensions of 10 cm x 10 cm and thicknesses of 25 mm and 40 mm were made. The density of the phantom was determined from measurements of the mass and volume of the two samples and was measured to be 1099.5 ± 18.0 kg/m^3^. Speed of sound and attenuation measurements were performed using a through-transmission substitution technique as described in detail in section 3 of Zeqiri et al. [23]. Two transducers were used in transmission to cover a wider range of frequencies, namely a 2.25 MHz unfocused PZT transducer (Panametrics-NDT, Olympus Industrial, Essex, UK) with an outer diameter of 32 mm, and a 5.3 MHz PVDF transducer (PA392, Precision Acoustics, Dorchester, UK) with an outer diameter of 23 mm. The detector used was a calibrated 0.5 mm PVDF needle hydrophone (PA2290, Precision Acoustics, Dorchester, UK). The hydrophone was placed aligned with the beam axis in the far-field region of the field generated by the transducer. Each transducer was driven with a single cycle burst at its centre frequency. Signals were acquired and digitised using an oscilloscope (DPO5034B, Tektronix UK Ltd., Berkshire, UK). The transmission through each of the samples and through the direct water path was measured. The FFT of the signals was obtained and the transmission loss through each sample was calculated. To remove the effect of interfacial losses, the attenuation coefficient was calculated from the ratio of the difference in transmission loss through the two samples to the difference in their thicknesses. Each set of measurements was repeated four times at different source - hydrophone distances, increasing in steps of 10 mm. The measured frequency-dependent attenuation coefficient was fitted with a power law of the form *α*=*α*_0_*f*^*y*^ over the frequency range from 1 to 9 MHz, where *α*_0_=0.1559 dB/cm/MHz^y^ and *y*=1.552 (R^2^=0.99). The attenuation measurements and power law fit are shown in Fig. 1. At the frequency of interest (3.3 MHz), the attenuation coefficient was ∼1 dB/cm, which is close to the previously reported values for the prostate [13, 24]. For the calculation of the speed of sound, the time of arrival of the pulses was determined from the time at which the signal at the beginning of the pulse was visible above the noise, with the acquisition synchronised with signal generation. The measured speed of sound at room temperature was 1550.5 ±1.5 m/s.

### Experimental setup

**Fig. 2:**
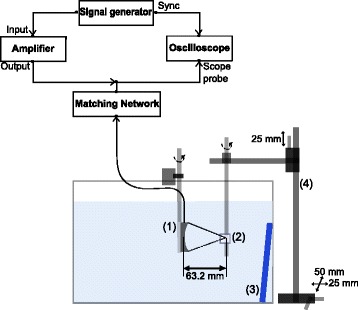
Schematic of experimental setup showing (1) the high-intensity focused ultrasound transducer, (2) phantom mount containing the tissue-mimicking material with implanted fiducial marker, (3) acoustic absorber used to prevent reflections, and (4) 3-axis manual positioning system

A schematic of the experimental setup is shown in Fig. 2. The experiments were performed in a scanning tank filled with deionized water at room temperature (∼24-26°C). The acoustic field was generated using a single-element spherically focused HIFU transducer (H-101, Sonic Concepts, Bothell, WA, USA) with an active diameter of 64 mm and focal length of 63.2 mm. The transducer was driven at 3.3 MHz (the third harmonic) using a continuous wave input signal generated by an arbitrary waveform generator (33522A, Agilent Technologies, PaloAlto, CA, USA) and amplified by an RF power amplifier (ENI A300, Electronics and Innovation, Rochester, NY, USA) before transmission to the transducer via an impedance matching network (H-101G Impedance Matching Network, Sonic Concepts, Bothell, WA, USA). The drive voltage was measured using a scope probe (N2863B, Agilent Technologies, PaloAlto, CA, USA) and an oscilloscope (DSO-X 3024A, Agilent Technologies, PaloAlto, CA, USA). The BSA PAG phantom was held within a custom PMMA mount and connected to a 3-axis manual positioning system with 0.01 mm positioning accuracy. An acoustic absorber was placed at the rear wall of the tank to prevent acoustic reflections. A peak-to-peak drive voltage of 112 V and a sonication time of either 10 s (single lesion experiments) or 6 s (raster scan experiments) was used in all reported experiments. These driving parameters were shown to create repeatable thermal lesions in preliminary measurements. The focal intensity was estimated using a combination of experimental acoustic holography and nonlinear modelling as reported in Kreider et al. [25]. In water, the estimated intensity was ≈1400 W/cm^2^, while in the BSA phantom, the estimated intensity was ≈1200 W/cm^2^. For comparison, the transducer in the clinically used Sonablate 500 (SonaCare Medical LLC, Charlotte, NC, USA) has a frequency of 4 MHz, element dimensions of 22 mm by 30 mm, two fixed focal lengths of 30 mm and 40 mm, and pulse duration 3 s [26]. The focal intensity reported ranges from 1000 to 2000 W/cm^2^ [27–29].

### Experimental protocol - single lesion

Two sets of experiments were performed. In the first set of experiments, the BSA PAG phantoms were cylindrical in shape, with a diameter of 20 mm and a length of 30 to 50 mm. Before sonication, each phantom was implanted with a single cylindrical marker (1 mm in diameter and 3 mm in length) made of stainless steel (*ρ*=8000 kg/m^3^, *c*=5970 m/s), which was used to replicate the gold EBRT fiducial markers used clinically. Due to a large number of experiments performed, it was cost prohibitive to use clinical markers for the experiments. However, the normal incidence pressure reflection coefficients of gold and stainless steel in prostate tissue are 0.95 and 0.93, respectively, thus the materials are acoustically very similar. The markers were inserted into the phantoms using a gauge 16 catheter needle (Catheter I.V., Insyte, 16 GA, 1.7 x 45 mm) modified with a copper wire used to push the markers out of the needle. A laser cut PMMA grid was used to allow precise alignment and needle orientation. The markers were always inserted at right angles to the beam axis. This orientation was chosen to mimic the most common clinical scenario where the markers are inserted transperineally, and the HIFU transducer is positioned transrectally. In some phantoms, there was a small amount of migration in the marker orientation when the catheter was removed, however, this was similar to the migration that occurs clinically. The phantom was then mounted in the tank with the cylindrical axis of the phantom aligned with the beam axis.

**Fig. 3:**
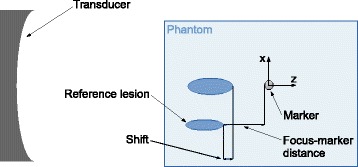
Schematic showing how the shift in lesion position and distance from the transducer focus to the marker were measured. Note, the schematic is not to scale; reference lesions were approximately 1 x 1 x 3 mm^3^

In each phantom, two lesions were created. First, a reference lesion was generated close to the edge of the phantom without the marker obstructing the HIFU beam path. Second, a lesion was generated with the transducer focus placed at a specified distance from the marker (see Fig. 3). For each phantom, the two lesions were always at the same depth, and sufficiently well separated to not affect the generation of the second lesion. The focus position was then systematically varied along either the anteroposterior or left-right axes relative to the marker in steps of 1 mm. In the anteroposterior direction (*z*-axis in Fig. 3), 31 different positions were tested, ranging from 10 mm in front of the marker to 20 mm behind it. These experiments were always conducted with the beam axis aligned with the marker (i.e., with no offset in the left-right or *x*-direction), and were repeated eight times. In the left-right direction (*x*-axis in Fig. 3), 11 positions were tested ranging from the centre of the marker to 10 mm to the side. These experiments were conducted for 16 different offsets in the anteroposterior direction, ranging from 5 mm in front of the marker to 10 mm behind. For this arrangement, the sonications at each of the positions were made only once.

After sonication, the volume and position of the produced lesions were measured to quantify the distortion introduced by the marker in comparison to the reference lesion in that phantom. The lesion dimensions and position were measured visually using a millimetre grid printed on transparent paper positioned in front of the phantom, with an uncertainty of ± 0.5 mm. The volume was then calculated assuming an ellipsoid, where $V=\frac {4}{3} \pi abc$ with *a*, *b*, and *c* being the measured semi-axes of the ellipsoid. The shift in lesion position was measured as the distance between the ends of the lesions distal from the transducer face, as shown in Fig. 3. The shift was attributed a positive value if the lesion position moved away from the transducer (in the positive z-dimension).

### Experimental protocol - raster scan

In the second set of experiments, the BSA PAG phantoms were cuboids in shape, with dimensions of 3×3×5 cm^3^. Again, each phantom was implanted with a single cylindrical marker as described in the previous section. The transducer was then raster scanned across the target region to create a larger ablation volume in order to mimic how the HIFU treatments are performed clinically. The raster scans were performed along the *x*-axis (left-right) for a fixed *z*-position relative to the marker, with 6 s on time, and 3 s off time. 21 different *z*-positions were tested, ranging from 10 mm anterior to the marker to 10 mm posterior. For each raster scan at a fixed *z*-position, 12 sonications were performed with the position of the focus for each sonication shifted by 1 mm in the *x*-direction. Raster scans at each of the *z*-positions were conducted once.

## Results

### Single lesion - offset along the anteroposterior axis

**Fig. 4:**
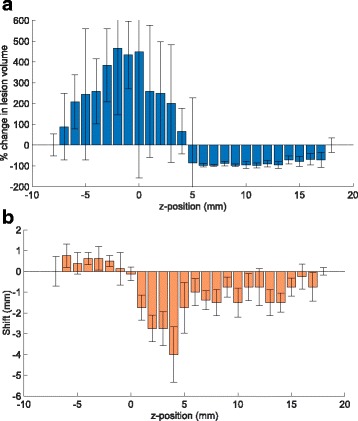
Change in the (**a**) lesion volume and (**b**) lesion position as a function of the offset between the focal position and the marker, when the focal position is translated along the anteroposterior (*z*) axis. Each measurement was repeated 8 times, and the error bars show one standard deviation. A negative change in volume indicates the lesion volume was reduced, and a negative shift indicates the lesion was shifted towards the transducer

Figure 4 shows the change in volume and position of the lesion as a function of the transducer focal position relative to the marker for an offset along the anteroposterior axis. For comparison, the volume of the reference lesion varied between 1×1×3 mm^3^ and 1×1×4 mm^3^, and always occurred at the same axial position. When the marker was positioned close to the transducer focus, the ultrasound beam was strongly aberrated, and the lesions produced differed in both size and position from the reference.

When the transducer focus was in front of the marker (negative *z*-position in Fig. 4), the impact of the marker on the lesion volume and position was noticeable up to an offset of -7 mm. The lesion increased in volume (sometimes more than four-fold) and was shifted closer to the marker due to reflections of the ultrasound energy from the marker. When the transducer focus was positioned at the marker, the lesion volume still increased due to reflection, but there was no shift in lesion position. When the transducer focus was positioned behind the marker (positive *z*-position in Fig. 4), the impact of the marker on the lesion volume and position was noticeable up to an offset of 18 mm. The lesion decreased in volume and was again shifted closer to the marker. This was due to a reduction in energy arriving at the intended focus because of scattering by the marker caused by the marker’s high reflectivity, as was previously mentioned. This is consistent with the simulation results presented in Georgiou et al. [13]. In the experiments, the shift in lesion position was especially prominent for focal positions up to 4 mm behind the marker, where the lesion was not produced at the desired position but was instead shifted in front of the marker.

**Fig. 5:**
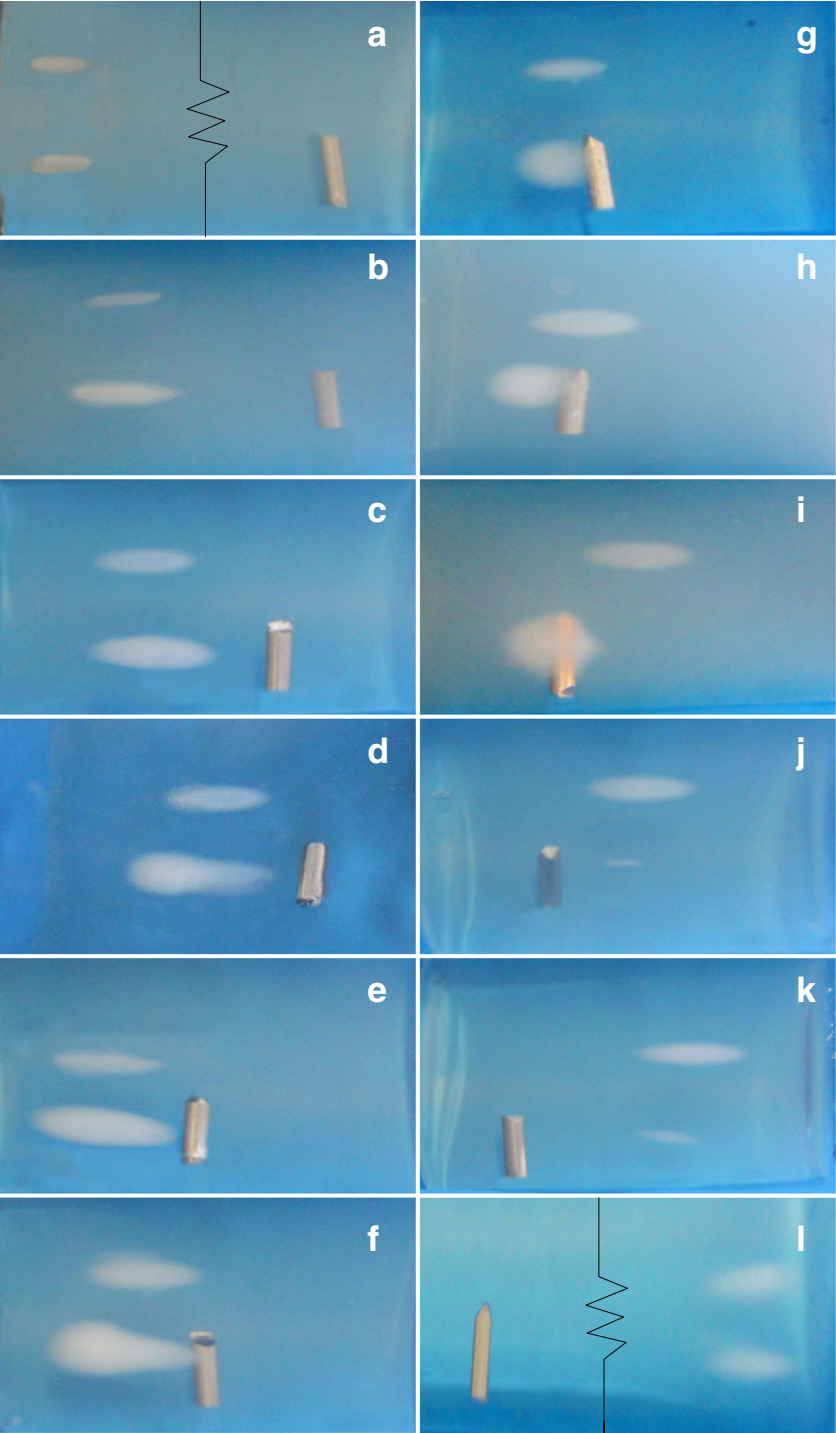
Lesions produced in BSA PAG phantoms with the transducer focus positioned 10, 5, 3, 2, 1 mm in front of the marker (**a**-**e**), at the marker (**f**) and 1, 2, 3, 5, 10, 20 mm behind the marker (**g**-**l**). The black broken line indicates that the photo was trimmed to fit both the marker and lesions

Examples of the lesions produced at different marker positions are shown in Fig. 5. Both the reference and distorted lesion are shown in each photograph. The lesions were produced with the transducer focus positioned 10, 5, 3, 2, 1 mm in front of the marker (a-e), at the marker (f), and 1, 2, 3, 5, 10, 20 mm behind the marker (g-l). The black broken line indicates that the photo was trimmed to show both the marker and lesions within the figure. As can be seen from the photos, the markers in each phantom were positioned at slightly different angles due to a slight migration of the marker during the insertion procedure.

### Single lesion - offset along the left-right axis

**Fig. 6:**
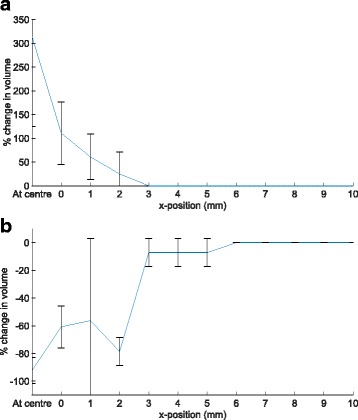
Change in the lesion volume as a function of the offset between the focal position and the marker, when the focal position is translated along the left-right (*x*) axis for a fixed position in the anteroposterior axis. The results are grouped for (**a**) the transducer focus positioned from 5 mm in front of the marker to 4 mm behind it (average of 10 measurements), and (**b**) the transducer focus positioned from 5 mm to 10 mm behind the marker (average of 6 measurements). The error bars show the standard deviation based on the grouped measurement results

Figure 6 shows the change in the lesion volume as a function of the transducer focal position relative to the marker for an offset along the left-right axis. Here, “at centre” corresponds to the transducer focus placed in the centre of the marker, and the zero position corresponds to the focus placed at the edge of the marker. Although these experiments were repeated for 16 different offsets in the anteroposterior direction, the results are collated into two groups based on the observed effect. As shown in Fig. 6a, there was an increase in the lesion volume when the transducer focus was positioned between 5 mm in front of the marker and 4 mm behind it, and within 3 mm of the edge of the marker in the left-right direction. A decrease in lesion volume was observed when the transducer focus was positioned between 5 mm and 10 mm behind the marker and within 6 mm of the marker edge. As the beam width at the marker position increased the further behind the transducer focus was placed, a larger offset was required between the focus and the marker in the *x*-direction to move the marker out of the beam path. These results, combined with the ones obtained in the first set of measurements, are in agreement with Georgiou et al. [13], where the simulations predicted the effect to be significant in an hourglass-shaped volume surrounding the fiducial marker.

### Raster scans

**Fig. 7:**
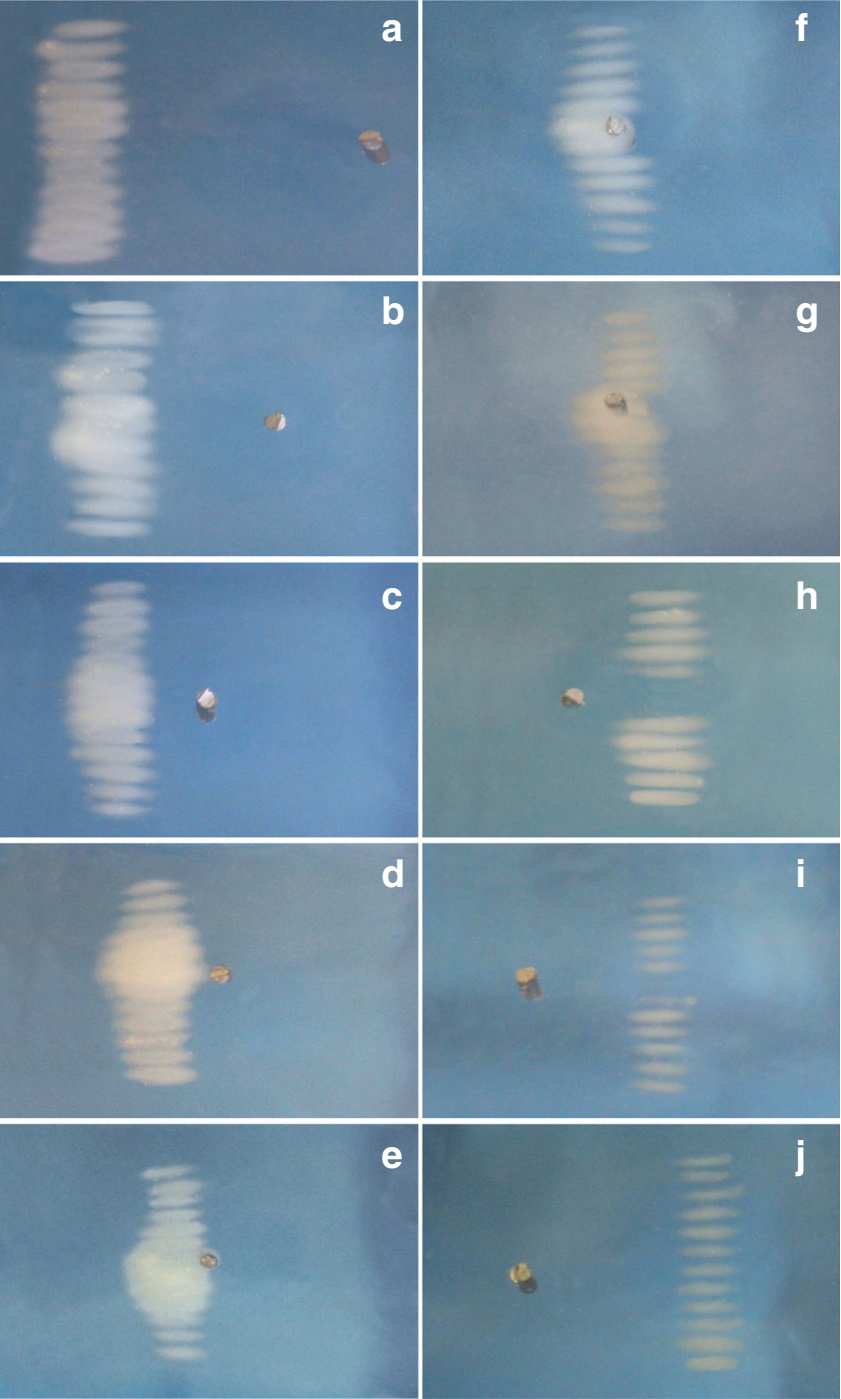
Lesions produced in BSA PAG phantoms after raster scanning the transducer in the *x*-direction with the transducer focus positioned 10, 5, 2, 1 mm in front of the marker (**a**-**d**), at the marker (**e**) and 1, 2, 5, 7, 10 mm behind the marker (**f**-**j**)

The experiment with the raster scans yielded results similar to the single lesion experiments, but the range of distances over which the effect was observed was slightly smaller. This was more evident with the transducer focus positioned behind the marker, where the effect diminished after 10 mm (compared to 17 mm in the single lesion experiment). The cause of this might be the reduced sonication time (6 s vs 10 s for the single lesion experiments), or that the neighbouring sonications partially increased the temperature in the shadow zone behind the marker, allowing a visible lesion to be generated even when the beam path was partially occluded by the marker. The results for several positions tested are shown in Fig. 7. When the transducer focus was between 5 mm in front and 2 mm behind the marker, the size of the ablated volume increased, with the ablation extending proximally to the marker (Fig. 7b-g). When the transducer focus was positioned further behind the marker, up to 7 mm, the ablated volume decreased, with a noticeable gap distal to the marker (Fig. 7h-i).

## Discussion

### Comparison with simulations

The experimentally measured changes in the lesion volume and position were similar to the changes reported in the simulation study presented in Georgiou et al. [13]. In particular, the lesion volume increased when the transducer focus was positioned immediately in front of the marker and reduced when positioned immediately behind. The predicted focal shifts were also of a similar magnitude (maximum shifts on the order of ∼5 mm), with the largest effect observed when the marker was within 6 mm along the anteroposterior axis. This suggests that the results are not strongly influenced by choices made in the experimental design which differ between the simulations and experiments.

**Fig. 8:**
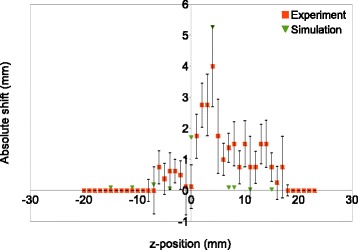
Comparison between the focal shift obtained in simulations and experiments. The error bars show the standard deviation of the experiment results

Changes in volume could not be quantitatively compared as in the numerical simulations only the -6dB focal volume of the temporal-average intensity was calculated (no thermal simulations were conducted), while in the measurements the ablation volume was measured. The change in position of the lesion centre obtained in the experimental measurements and the distance between the intended focus and the coordinates of the maximum pressure point from the simulations are shown in Fig. 8. Both simulations and experiments give similar values for the magnitude and range of the effect of the marker. As an example, the biggest change in position occurs with the transducer focus positioned 4 mm behind the marker, with a shift of 4-5 mm.

### Transducer geometry

**Fig. 9:**
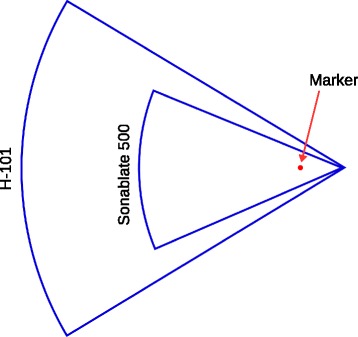
Schematic showing the effect different transducer geometries have on the portion of the HIFU beam obstructed by the marker

While the experimental results replicate the numerical predictions, some limitations of this work are as follows. Firstly, the HIFU transducer used in the experimental study differs geometrically from the transducers used clinically. For example, lesions generated with no markers present using the Sonablate 500 probe are significantly larger than the lesions produced by the H-101 transducer used here (3×3×10 mm^3^ vs 1×1×3 mm^3^). However, as the aperture for the Sonablate 500 transducer is smaller (see Fig. 9), this means that proportionally, the marker will interact with a larger portion of the HIFU beam. This suggests the aberrations may be worse in the clinical scenario compared to the results presented here, although the simulations using the clinical geometry, despite not modelling the thermal aspect, show comparable effects.

Second, for clinical systems, cavitation effects also play a role in tissue destruction, while only thermal lesions have been considered here. While the reduction in focal intensity due to the marker will likely also reduce the onset and magnitude of cavitation activity, it is difficult to infer exactly how this will relate to the results obtained in this work.

### Measurement uncertainties

Considering measurement uncertainties, the markers in each phantom were positioned at slightly different angles due to migration after insertion. This could have influenced the size of the observed effect for any given experiment, although it is unlikely to change the overall distortion introduced by the marker. Also, the lesion volume was quantified assuming it had an ellipsoidal shape, which may introduce errors for some lesion shapes (e.g., see Fig. 5). Finally, only one orientation of the marker relative to the HIFU beam was investigated. For other orientations (e.g., with the long axis of the marker aligned with the beam axis), a different magnitude of the effect might be observed.

## Conclusion

The effect of a single EBRT fiducial marker on the efficacy of HIFU treatment delivery was experimentally investigated using a tissue-mimicking phantom and a single element HIFU transducer. A modified recipe for a prostate TMM was developed based on a polyacrylamide hydrogel containing bovine serum albumin. The acoustic properties of the TMM were measured and shown to be similar to the properties of the prostate. A series of experiments were then conducted to investigate the effect of the marker on the size and volume of the generated lesion when the transducer focus is positioned close to the marker. The results demonstrate that the markers can significantly distort the shape, position, and size of a HIFU lesion when positioned close to the focus. This may result in an undertreated region beyond the marker due to a reduction in energy arriving at the focus, and an overtreated region in front of the marker due to reflections. Depending on the position of the targeted regions and the distribution of the markers, both effects may be undesirable and may affect treatment efficacy. Further work is needed using clinically available prostate HIFU devices to assess the importance of these observations in clinical practice and to assess whether it is necessary to reconsider patient selection and treatment planning for prostate salvage HIFU after failed EBRT. Whilst not explicitly studied, these results are also relevant for salvage HIFU following brachytherapy, where a large number of seeds (acoustically similar to the gold EBRT fiducial markers) are implanted in the prostate.

## Abbreviations

APS: Ammonium persulfate
BSA: Bovine serum albumin
EBRT: External beam radiation therapy
HIFU: High-intensity focused ultrasound
IGRT: Image guided radiotherapy
PAG: Polyacrylamide hydrogel
PMMA: Poly(methyl methacrylate)
PVDF: Polyvinylidene fluoride
PZT: Lead zirconate titanate
TEMED: Tetramethylethylenediamine
TMM: Tissue-mimicking material
TRIS: Trisaminomethane
